# Molecular and Thermodynamic Insights into the Enthalpy-Entropy Shift Governing HILIC Retention of Labelled Dextrans

**DOI:** 10.3390/molecules30244711

**Published:** 2025-12-09

**Authors:** Matjaž Grčman, Črtomir Podlipnik, Matevž Pompe, Drago Kočar

**Affiliations:** Faculty of Chemistry and Chemical Technology, University of Ljubljana, Večna pot 113, SI-1000 Ljubljana, Slovenia; matjaz.grcman@fkkt.uni-lj.si (M.G.); crtomir.podlipnik@fkkt.uni-lj.si (Č.P.); matevz.pompe@fkkt.uni-lj.si (M.P.)

**Keywords:** HILIC, dextran ladder, retention mechanism, enthalpy–entropy compensation

## Abstract

Hydrophilic interaction liquid chromatography (HILIC) is widely used for the analysis of glycans and oligosaccharides, yet the molecular basis of retention remains incompletely understood. In this study, we investigated dextran ladders labelled with 2-aminobenzamide (2-AB) and Rapifluor-MS™ (Waters, Milford, MA, USA) across a wide range of degrees of polymerization (DP 2–15), temperature conditions (10 °C to 70 °C), and gradient programs using a Acquity™ Premier Glycan BEH Amide column (Bridged Ethylene Hybrid, Waters, Milford, MA, USA). Van’t Hoff analysis revealed distinct enthalpic and entropic contributions to retention, allowing identification of a mechanistic transition from enthalpy-dominated docking interactions at low DP to entropy-driven dynamic adsorption at higher DP. This transition occurred reproducibly between DP 4–6, depending on the fluorescent label, while gradient steepness primarily influenced the location of the minimum enthalpy. Molecular dynamics simulations provided additional evidence, showing increased conformational flexibility and end-to-end distance variability for longer oligomers. This finding is consistent with entropy-dominated adsorption accompanied by displacement of structured interfacial water. Together, these results establish a molecular-level framework linking retention thermodynamics, conformational behavior, and solvation effects, thereby advancing our mechanistic understanding of glycan separation in HILIC.

## 1. Introduction

Hydrophilic interaction liquid chromatography (HILIC) has become an essential tool for analyzing biomolecules, including oligosaccharides and glycans [1,2]. The central role of water in this mechanism arises from its unique molecular structure, which leads to characteristic thermodynamic properties during solvation and reorganization of its hydrogen-bonding network [3]. The retention mechanism in HILIC is generally attributed to the presence of a water-rich layer surrounding the polar stationary phase, enabling partitioning and adsorption of analytes through a combination of hydrogen bonding, dipole–dipole, and electrostatic interactions [4]. However, the complexity of these processes often results in competing and shifting mechanisms, with the dominant mode of retention changing depending on solvent composition, analyte size, and conformational behavior [5]. In particular, studies of silica-based stationary phases have shown that surface silanol groups stabilize a structured water layer, where water molecules form an ordered hydrogen-bonding network at the solid–liquid interface. When an analyte enters this interfacial region, its polar groups can interact with and locally disrupt the ordered water structure. This disruption not only promotes adsorption through docking-type interactions but also alters the enthalpic and entropic contributions to retention, assigning the structured water layer a central role in HILIC thermodynamics [6,7,8,9].

In our previous study [9], we systematically examined the separation of fluorescently labelled dextran ladders on a Acquity™ Premier Glycan BEH Amide column (Bridged Ethylene Hybrid) and evaluated the effect of mobile phase conditions on retention and selectivity. We found that increasing ionic strength (10–100 mM ammonium formate, pH 4.4) had only a minor influence on retention, whereas the use of methanol-based mobile phases led to pronounced changes in selectivity [10]. Comparisons of different eluents showed that acetonitrile promotes the formation of a more extensive and stable adsorbed water layer, while methanol partially disrupts or competes with interfacial water, resulting in a thinner layer and weaker partitioning contributions [11]. These findings indicate a change in the underlying retention mechanism, raising the question of which molecular and thermodynamic processes are responsible for the observed behavior.

In this study, we extend our investigation by conducting a thermodynamic analysis of retention. Using van’t Hoff evaluation across multiple temperatures and gradient conditions, we determined the enthalpic (Δ*H*) and entropic (Δ*S*) contributions and calculated the Gibbs free energy of retention (Δ*G*). This allowed us to decompose the driving force of retention into enthalpic and entropic terms (Δ*G* = Δ*H* − *T*Δ*S*), providing deeper mechanistic insight into how methanol modulates retention processes [12,13].

To complement the thermodynamic analysis, we also performed computational modelling of analyte structures. Minimum energy conformations of labelled dextrans in water were first obtained using Spartan with appropriate basis sets. These conformations were then used as input for molecular dynamics (MD) simulations in Yasara [14,15], where we monitored parameters such as the hydrodynamic radius and the end-to-end distance between the terminal atoms of the fluorophore tag and the sugar chain. These structural descriptors provided additional context for interpreting the experimental retention behavior, offering a molecular-level view of how conformational flexibility and solvent interactions contribute to enthalpy–entropy compensation.

Another aspect considered in this study is the role of oligosaccharide size and conformational flexibility. Previous studies have shown that polysaccharides can undergo solvent-dependent conformational changes, with hydrogen bonding and cohesive solvent forces influencing the degree of coil expansion or contraction [16]. These structural effects add another dimension to retention behavior, particularly at higher degrees of polymerization, where chain flexibility and solvent interactions are amplified [17].

To rationalize our findings, we interpret the results within the framework of preferential hydration and water displacement [18,19,20]. In this context, the BEH Amide stationary phase can be viewed as a hydrated interface where water molecules are either preferentially retained or displaced by methanol. The balance between these processes determines the magnitude and sign of enthalpic and entropic contributions to retention, thereby explaining the observed selectivity changes in methanol-based gradients.

## 2. Results and Discussion

In order to systematically investigate these thermodynamic contributions in HILIC separation, we designed a study using dextran ladders as model oligosaccharides. The obtained results are additionally supported by simulation-derived metrics (Kirkwood hydrodynamic radius and end-to-end distance, Table 1). Two different fluorescent labels were used: RapiFluor-MS™ and 2-aminobenzamide (2-AB), both commonly used in glycan analysis [21,22]. The experiments were performed with a BEH Amide stationary phase [23], under conditions specifically chosen to probe methanol as the organic modifier.

Two gradient programs were used: a flatter gradient (gradient 1, Table 2) and a steeper gradient (gradient 2, Table 3), where mobile phase A consisted of 95% methanol and 5% water with 20 mM ammonium formate (pH 4.4), and mobile phase B was 5% methanol and 95% water with the same buffer concentration. This setup ensured that ionic strength and pH remained constant across all conditions, while the proportion of methanol served as a systematic variable for probing retention behavior.

**Figure 1:**
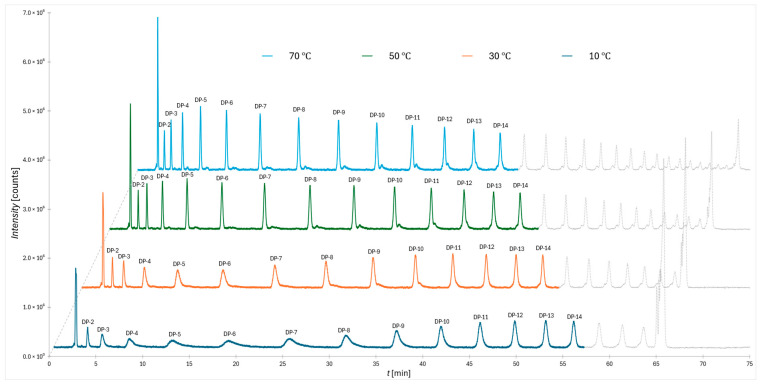
Chromatograms of 2-AB-labeled dextrans (flatter gradient) at different column temperatures.

In Figure 1 the chromatograms obtained for the 2-AB labeled dextran ladder under a flatter gradient at four column temperatures (10 °C, 30 °C, 50 °C, and 70 °C) are shown. It reveals a systematic temperature effect on separation quality. Within the applied gradient, oligomers from DP 2 to DP 14 were resolved, with the most prominent distortions observed at the lowest column temperature. Specifically, the peaks appear broadened, asymmetric, and reduced in intensity, reflecting a clear decrease in separation efficiency. This deterioration provides strong evidence for a temperature-dependent shift in the balance of retention mechanisms, most notably between adsorption through water displacement at the stationary phase surface and the additional solvation-related contribution described previously in the literature [24].

It should be emphasized that the same gradient conditions also allow full resolution up to DP 14 for the RapiFluor-MS™ labeled dextran ladder, supporting the robustness of the approach across different fluorescent tags. In all experiments, column preconditioning was applied for both selected gradients to ensure equilibration of the stationary phase prior to analysis, thereby minimizing baseline drift and enhancing reproducibility (as shown in Table 2 and Table 3).

By combining these experimental conditions with van’t Hoff analysis, we aimed to clarify the thermodynamic basis of the selectivity changes observed in methanol-containing HILIC separations of dextrans. The retention behavior in HILIC systems can be quantitatively described using by changes in free energy, which is composed of entropy and enthalpy changes.

(1) $$-RT\ln k^{\prime }=∆G-RTln\Phi$$

where Φ represents phase ratio, that is ratio between volume of stationary phase and volume of mobile phase. Based on previous studies on amide-type HILIC columns, which reported a phase ratio of Φ ≈ 0.08 in acetonitrile/water systems and a water-layer thickness of about 0.58 nm corresponding to one to two molecular layers of water. Further investigations into the influence of methanol as an organic modifier have shown that it markedly reduces the amount of adsorbed water on polar stationary phases. Even small additions of MeOH disrupt the formation of structured multilayer water films, leading to a thinner and more dynamic interfacial layer [25,26]. Given that the eluent used in this work contained methanol as the organic modifier, it is therefore reasonable to assume that only a single, tightly bound water layer remains adsorbed on the amide surface. Since such a monolayer structure is expected to exhibit only limited structural reorganization, we may reasonably assume that its thickness and composition are only weakly affected by temperature, and thus the phase ratio (Φ) can be considered approximately temperature-independent. Consequently, the effective phase ratio of Φ = 0.04 was used in this study.

Using van’t Hoff approach, which relates the logarithm of the retention factor (*k*) to the reciprocal of absolute temperature (1/*T*). According to the linear form of Equation (2):

(2) $$\ln k^{\prime }=-\frac{∆H}{R}\cdot \frac{1}{T}+\frac{∆S}{R}+ln\Phi$$

Therefore, the slope of the van’t Hoff plot yields the enthalpic contribution to retention (Δ*H*), while the intercept reflects the entropic term (Δ*S*) and phase ratio. From these parameters, the Gibbs free energy (Δ*G*) can be derived as Δ*G* = Δ*H* − *T*Δ*S*, providing a comprehensive view of the thermodynamic driving forces governing retention. This framework enables us to separate and interpret the relative importance of enthalpy- and entropy-driven interactions in complex HILIC systems.

**Figure 2:**
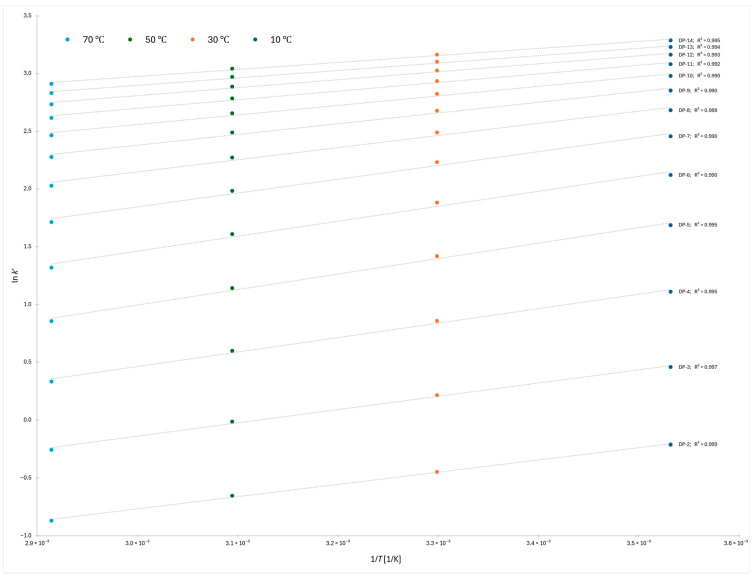
van’t Hoff plot of 2-AB labeled dextrans from DP 2 to DP 14 (flatter gradient).

The van’t Hoff plots constructed for the 2-AB labeled dextran ladder across the four column temperatures (10 °C, 30 °C, 50 °C, and 70 °C) showed highly consistent linearity over the oligomer range DP 2–14 (Figure 2). For each degree of polymerization, a robust linear fit was obtained, with correlation coefficients (R^2^) exceeding 0.988 in all cases. This indicates that the retention behavior of the dextran oligomers can be reliably described by a two-parameter thermodynamic model, allowing direct extraction of enthalpic and entropic contributions to retention. The uniformity of the fits across the series further supports the reproducibility of the applied gradient and equilibration strategy and highlights the suitability of van’t Hoff analysis for quantitatively probing the molecular mechanisms governing retention in HILIC systems.

**Figure 3:**
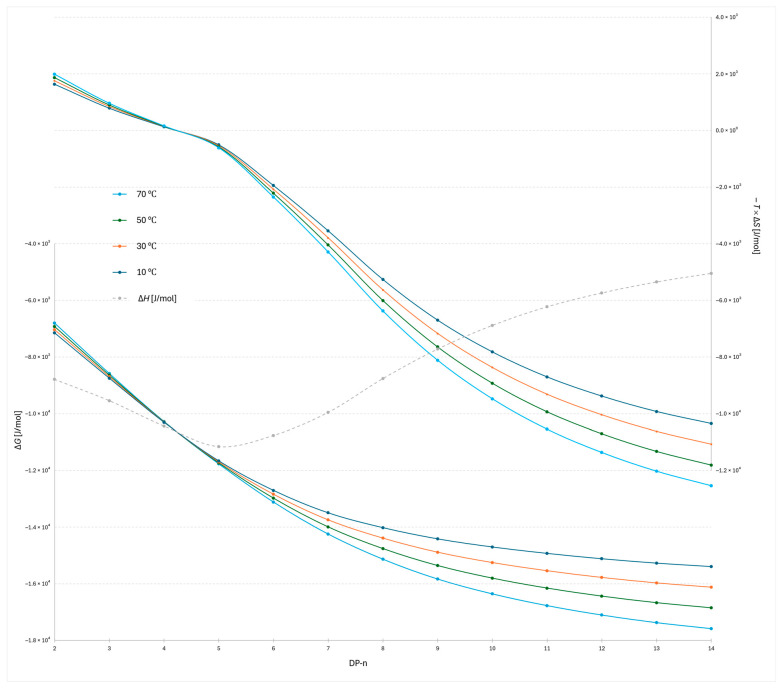
Plots of Δ*G* and −*T*Δ*S* versus degree of polymerization (DP 2–14) for the 2-AB labeled dextran ladder under four column temperatures (10 °C, 30 °C, 50 °C, and 70 °C) obtained with the flatter gradient.

The plot of −*T*Δ*S* versus degree of polymerization for the 2-AB labeled dextran ladder (Figure 3) provides detailed insight into the entropic contribution to retention. For the lowest oligomers, DP 2–5, the data indicate that higher temperatures (70 °C) are less favorable in terms of Gibbs free energy, as reflected by the largest positive −*T*Δ*S* contributions. This suggests that retention of the smallest oligomers occurs through a docking-type interaction at the stationary phase surface, characterized by strong, oriented hydrogen bonding. The high −*T*Δ*S* values observed for these oligomers are therefore consistent with entropically unfavorable processes, as docking reduces molecular disorder during retention. Correspondingly, the Δ*H* values are strongly negative, indicating the formation of stabilizing interactions that enthalpically compensate for the entropic penalty. This behavior is consistent with broader observations on oligosaccharide interactions, where the dominant entropic barrier arises from the loss of conformational degrees of freedom during binding, while enthalpic stabilization is provided by specific, oriented hydrogen bonds [17,25]. A similar principle has also been highlighted in molecular docking studies of flexible ligands, where hydrogen bonding and limited flexibility strongly influence the free energy of binding, which is conceptually similar to this study [26].

A distinct transition is observed between DP 4 and 5, where the relative temperature dependence inverts (Δ*S* = 0): above this point, higher temperatures become more favorable for stronger retention, as evidenced by lower −*T*Δ*S* values compared to the lower-temperature profiles. This transition highlights a mechanistic shift from docking-driven interactions dominating at low DP to a regime at higher DP where dynamic adsorption and conformational effects govern retention. This behavior can also be explained in terms of the structured interfacial water layer: while small oligomers interact locally with the ordered network, larger oligomers disrupt and displace it more extensively, and for the longest oligomers this results in a greater entropic contribution, consistent with reported simulations of layered water structures at silica interfaces and reflected by the lowest −*T*Δ*S* values [6,27]. The combined Δ*H* and −*T*Δ*S* data thus emphasize that small oligomers are retained through highly oriented, entropically unfavorable binding, whereas longer oligomers engage in more flexible, dynamic interactions that benefit from elevated temperature. This is consistent with reports that higher temperature reduces the rigidity of the adsorbed water layer [28], allowing solute molecules to move more freely within this interfacial region and leading to more dynamic adsorption.

**Figure 4:**
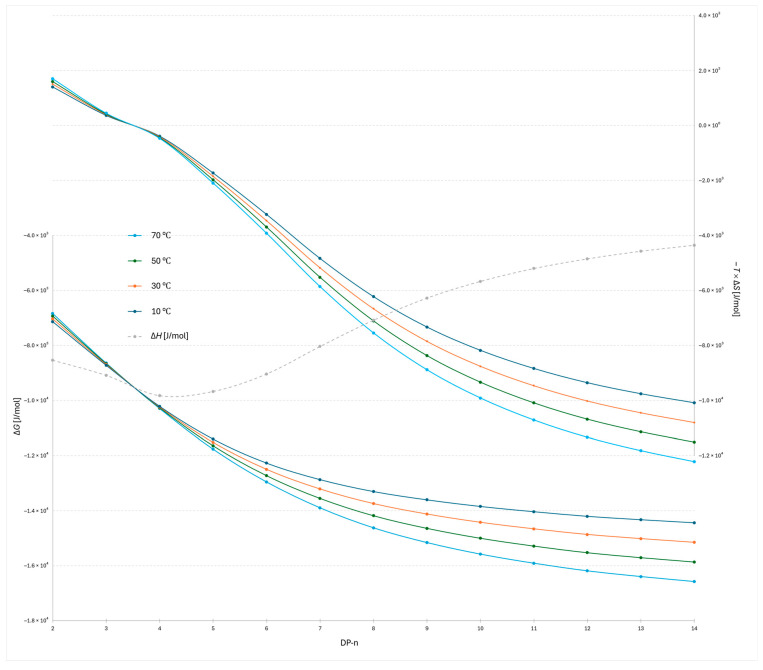
Plots of Δ*G* and −*T*Δ*S* versus degree of polymerization (DP 2–14) for the 2-AB-labeled dextran ladder at four column temperatures (10 °C, 30 °C, 50 °C, and 70 °C) obtained with the steeper gradient.

The plot of −*T*Δ*S* versus degree of polymerization for the 2-AB labeled dextran ladder under the steeper water gradient reveals both similarities and distinct differences compared to the flatter gradient (Figure 3 and Figure 4). The most evident similarity is that the transition point between entropic favorability at different temperatures again occurs between DP 3 and 4, confirming the robustness of this mechanistic transition. However, notable differences are observed in the enthalpic profiles: the lowest Δ*H* value in the steeper gradient is detected at DP 4, whereas in the flatter gradient the corresponding minimum occurs at DP 5. This shift indicates that gradient steepness modulates the balance of adsorption and docking interactions for the smallest oligomers, slightly altering the position of maximal enthalpic stabilization while leaving the overall mechanistic transition unchanged.

**Figure 5:**
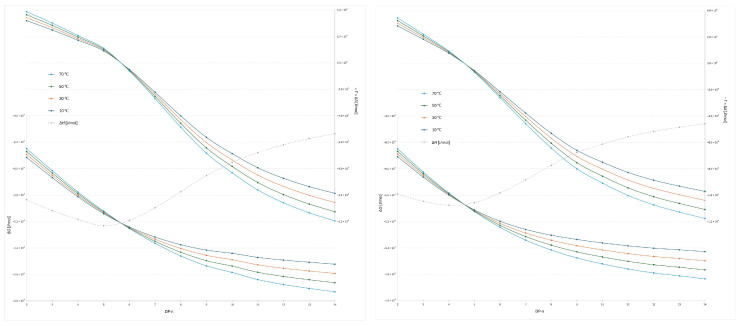
Plots of Δ*G* and −*T*Δ*S* versus degree of polymerization (from DP–2 to DP–14) for the RapiFluor-MS™ labeled dextran ladder under four column temperatures (10 °C, 30 °C, 50 °C, and 70 °C) obtained with the flatter gradient (**left**) and steeper gradient (**right**), respectively.

In Figure 5, where the RapiFluor-MS label was used, the transition (Δ*S* = 0) occurs between the same oligomers (between DP 5 and 6 using flatter gradient and between DP 4 and 5 using steeper gradient). The enthalpy trend is similar to that observed with the 2-AB label; however, with the 2-AB label, the transition occurs between DP 3 and 4, regardless of the gradient used. The minimum Δ*H* value is reached at DP 4 under the steeper gradient and at DP 5 under the shallower gradient. Notably, the position of the transition shifts compared to the previous experiments (Figure 3 and Figure 4), indicating that the transition point is probe-dependent but gradient-independent. Conversely, the location of the minimum Δ*H* is determined by both the DP and the gradient but remains unaffected by the type of probe used.

A temperature-dependent transition from enthalpy- to entropy-dominated retention emerges as oligomer size increases. For low DP (≲5), van’t Hoff behavior indicates predominantly enthalpic anchoring: Δ*H* is markedly negative, while Δ*S* is also negative, making −*T*Δ*S* positive. In this regime, lowering the temperature strengthens retention (more favorable Δ*G* via Δ*H*), while heating weakens it. At an intermediate size (DP between 3 and 6), the apparent entropy term crosses zero (Δ*S* ≈ 0), marking a chromatographic retention-mechanism transition. This separation regime shift reflects a change from enthalpy-driven docking interactions to entropy-driven dynamic adsorption. For higher DP (≥6), conformational dynamics and water release at the interface render Δ*S* > 0, so −*T*Δ*S* becomes increasingly negative with temperature and begins to dominate Δ*G* = Δ*H* − *T*Δ*S*. Consequently, raising the temperature enhances retention for large oligomers (entropy-driven regime), consistent with the observed inversion of temperature trends and the elevated conformational variability (large RSD in end-to-end distance) seen for DP 11 and above. Importantly, this mechanistic transition occurs reproducibly but with probe-dependent positioning: in 2-AB labeled dextrans, the transition is observed at DP around 4, whereas in the RapiFluor-labeled series, it shifts one unit higher, at DP around 5. Similarly, gradient steepness affects both the location of the minimum Δ*H* (DP 4 for steeper gradients, DP 5 for shallower), and the transition position itself. However, the transition position happens in all cases around Δ*H*_min_. The difference in and apparent transition position is less than 1 DP unit, which can also indicate, that the phase ratio has to be taken into account and its value is reasonable. These findings demonstrate that while the enthalpy-to-entropy transition is an intrinsic feature of the oligosaccharide size scale, its precise onset is modulated by the chemical nature of the label, whereas the gradient primarily influences the balance of adsorption versus docking interactions for the smallest oligomers.

The simulation-derived metrics (Kirkwood hydrodynamic radius and end-to-end distance) for 2-AB labeled oligomers at 10 °C and 70 °C strongly support the thermodynamic picture inferred from the Van’t Hoff analysis (Table 1). For smaller oligomers (e.g., DP 3 and DP 6), the average values show only minor temperature dependence within experimental uncertainty, and the RSD remains low. This is consistent with a “docking” mode at the hydrated BEH-amide surface, where directional hydrogen bonds generate strongly negative enthalpy, while the associated ordering of interfacial water leads to negative entropy contributions. Consequently, Δ*G* is positive and becomes less favorable with increasing temperature. In contrast, for DP 11, a pronounced increase in the RSD of the end-to-end distance indicates entry into a highly flexible conformational regime with a large number of accessible microstates. Such conformational plasticity facilitates dynamic adsorption, where continuous rearrangement of contact segments efficiently displaces structured interfacial water. As a result, entropy increases (becomes less negative or positive), and at 70 °C it becomes markedly more negative, providing an entropic driving force for retention. The observed rise in entropic favorability beyond the conformational “transition” (around DP 4 to 6) fully agrees with our earlier findings on a mechanistic transition: from enthalpy-dominated binding at low DP to entropy-driven, dynamic adsorption at higher DP. This explains why elevated temperature promotes retention of longer oligomers by enhancing the negative contribution to the overall free energy of adsorption.

## 3. Materials and Methods

### 3.1. Chemicals

Methanol (ULTRA Gradient HPLC Grade) was purchased from J. T. Baker (Gliwice, Poland). Ammonium formate (Sigma-Aldrich, for LC-MS LiChropur™, ≥99.0%, St. Louis, MO, USA) and formic acid (Fluka, puriss p.a., ≥98%, Muskegon, MI, USA) were used to prepare the buffer solutions. Ultra-pure water with a resistivity of 18 megohms per centimeter, produced by a Millipore Synergy^®^ UV-R purification system (Millipore, Burlington, MA, USA), was used.

We used two fluorescently labeled dextran samples-standards from Waters: RapiFluor-MS™ Dextran Calibration Ladder (50 μg/vial, P/N: 186007982, L/N: 0126132761) and 2-AB Dextran Calibration Ladder (200 μg/vial, P/N: 186006841, L/N: W28082324). Both samples were reconstituted in water as recommended by the manufacturer.

### 3.2. Instrument

The analytical setup used was the Thermo Scientific Vanquish Flex UHPLC instrument equipped with a binary pump and a fluorescence detector (UltiMate FLD 3400 RS) UHPLC instrument and fluorescence detector were purchased from Thermo Fisher Scientific (Waltham, MA, USA).

For all analyses, a Waters Acquity™ Premier Glycan BEH Amide column (2.1 × 150 mm, 1.7 µm, 130 Å; P/N: 186009976, S/N: 01763208815301, Waters, Milford, MA, USA) was installed on the chromatograph. The sample injection volume was set at 1.0 µL to ensure high analytical precision and sensitivity.

The fluorescence detector for the RapiFluor-MS™ Dextran calibration ladder sample was set with excitation at 265 nm and emission at 425 nm, while for the 2-AB dextran calibration ladder, the excitation wavelength was set to 330 nm and emission at 420 nm. A sampling rate of 10 Hz was used for data collection. The chromatographic data were processed and analyzed using Thermo Scientific Qual Browser.

### 3.3. Measurement

Chromatographic measurements were performed using an equilibrated chromatographic system. A constant buffer concentration was used in both mobile phases (20.0 mM ammonium formate, pH 4.4). Mobile phase A consisted of 5% MeOH and 95% aqueous phase, while mobile phase B consisted of 95% MeOH and 5% aqueous phase. A description of the applied gradient conditions, mobile phase composition, and additional chromatographic parameters is provided in Table 2 and Table 3.

**Table 2:** Gradient 1. Equilibration stage is marked at the beginning of the table with negative time.

Time [min]	Flow [mL/min]	Mobile Phase A [%]	Mobile Phase B [%]
−1.8	0.2	4.7	95.3
0	0.2	5.6	94.4
60	0.2	35.6	64.4
61.5	0.2	100	0
64.5	0.2	100	0
68.1	0.2	5.6	94.4
72.6	0.2	5.6	94.4
90	0.2	5.6	94.4

**Table 3:** Gradient 2. Equilibration stage is marked at the beginning of the table with negative time.

Time [min]	Flow [mL/min]	Mobile Phase A [%]	Mobile Phase B [%]
−1.8	0.2	4.1	95.9
0	0.2	5.6	94.4
60	0.2	55.6	44.4
61.5	0.2	100	0
64.5	0.2	100	0
68.1	0.2	5.6	94.4
72.6	0.2	5.6	94.4
90	0.2	5.6	94.4

Experiments were performed at 4 different column temperatures (10 °C, 30 °C, 50 °C and 70 °C, respectively).

### 3.4. Computational Modeling

Pre-optimisation of substrates was performed using SPARTAN’24 Parallel Suite (Wavefunction) for geometry pre-optimisation, applying the semi-empirical method (PM3). The geometries were then re-optimised for relevant equilibrium structures using Gaussian 16, applying the wB97X-D/6-31G*/SMD (water) density functional with the 6-31G* basis set as implemented in Gaussian 16. Normal mode vibrational analysis of the stationary points confirmed that they are minima (zero imaginary frequencies).

The optimized geometries served as initial structures for molecular dynamics (MD) simulations. Simulations were performed in Yasara (v24.15.5) using the AMBER14 force field, with GAFF parameters automatically applied to the nonprotein residues [14,15,29]. Systems were placed in a periodic cubic simulation cell with 15 Å padding around the solute and explicit water. Long-range electrostatics were treated with particle-mesh Ewald, van der Waals interactions with an 8 Å cutoff. Temperature control used velocity rescaling, while pressure and density control used Solvent Probe with water density fixed at 0.999 g mL^−1^ at 283 K. Each system was energy-minimized and then simulated at 283 K and 343 K for approximately 200 ns per condition. To allow the initially minimized Spartan structures to relax into an equilibrated state, the first 50 ns of each trajectory were discarded; all structural analyses were performed on the remaining 150 ns of production data. From these equilibrated frames, we computed the hydrodynamic radius using the Kirkwood–Riseman relation and the end-to-end distance between the terminal atom of the fluorophore tag and the terminal sugar unit. These descriptors were correlated with experimental thermodynamic parameters to contextualize how conformational flexibility and solvent interactions contribute to enthalpy–entropy behavior in HILIC.

Post-processing of Yasara trajectories was performed using a custom Python script (v 3.12) to analyze the simulation data, including calculation of the hydrodynamic radius and the end-to-end distance between the terminal atom of the fluorophore tag and the terminal sugar unit. All results were averaged and are reported with relative standard deviations (RSD, %) as a measure of variability.

## 4. Conclusions

In this work, we combined thermodynamic analysis of van’t Hoff plots with molecular metrics from simulations to elucidate the retention mechanism of dextran oligomers in HILIC on BEH-amide phases. By examining both 2-AB and RapiFluor MS-labeled ladders across a wide range of degrees of polymerization, from DP 2 to DP 15, as well as varying gradients and temperatures, we demonstrated that the retention mechanism undergoes a distinct molecular transition. For short oligomers, adsorption is enthalpy-dominated, driven by directional hydrogen bonding and accompanied by entropically unfavorable ordering of interfacial water. As DP increases, a mechanistic transition occurs, coinciding with the onset of greater conformational flexibility. This transition is evident in the cubic dependence on DP and in the increase in the RSD of end-to-end distances at intermediate chain lengths. For higher oligomers, dynamic adsorption becomes the dominant mechanism: conformational plasticity enables continuous rearrangement of contact points, which facilitates displacement of structured water at the surface. The associated entropic gain explains the emergence of increasingly negative values at elevated temperatures, providing the thermodynamic basis for stronger retention of larger oligomers under these conditions.

Taken together, our results establish a molecular-level framework that links retention thermodynamics with conformational behavior and interfacial water structuring. This framework rationalizes the observed transition from enthalpy- to entropy-driven adsorption and provides a mechanistic basis for predicting temperature- and DP-dependent retention behavior of glycans in HILIC. Beyond its immediate analytical relevance, this work highlights how combined experimental and molecular-level approaches can disentangle the complex interplay of solvation, adsorption, and conformational dynamics, offering new perspectives for advancing glycan separations and understanding HILIC retention mechanisms.

## Figures and Tables

**Table 1:** Hydrodynamic radii (K-R), end-to-end distances (E-E), and corresponding RSD values for selected dextran oligomers (DP 3, 6, and 9) with a 2-AB tag at 10 °C and 70 °C, serving as molecular descriptors in the thermodynamic analysis of HILIC retention.

	DP n	Kirkwood-Riseman Radius (K-R), Å	RSD (K-R), %	End-to-End (E-E) Distance, Å	RSD (E-E), %
10 °C	DP 3	6.7	1.6	20.1	13
	DP 6	7.9	5.2	18.8	16
	DP 11	8.6	2.4	15.9	36
70 °C	DP 3	6.7	1.9	18.6	19
	DP 6	7.8	5.7	17.8	21
	DP 11	8.5	2.3	11.3	48

## Data Availability

The original contributions presented in the study are included in the article, further inquiries can be directed to the corresponding author.
